# Pre-Existing Lymphopenia Increases the Risk of Hospitalization and Death after SARS-CoV-2 Infection

**DOI:** 10.3390/idr14010003

**Published:** 2022-01-04

**Authors:** Riccardo Garbo, Francesca Valent, Gian Luigi Gigli, Mariarosaria Valente

**Affiliations:** 1 Clinical Neurology Unit, Department of Neurosciences, Santa Maria della Misericordia University Hospital, 33100 Udine, Italy; gianluigi.gigli@uniud.it (G.L.G.); mariarosaria.valente@uniud.it (M.V.); 2 Clinical and Evaluational Epidemiology Service, Department of Governance, Local Health Authority, 38123 Trento, Italy; francesca.valent@apss.tn.it; 3 Department of Medical Area (DAME), University of Udine, 33100 Udine, Italy

**Keywords:** SARS-CoV-2, COVID-19, lymphopenia, lymphocytes

## Abstract

There is limited information regarding the severity of COVID-19 in immunocompromized patients. We conducted a retrospective cohort study considering the period from 1 March 2020 to 31 December 2020 to determine whether previously existing lymphopenia increases the risk of hospitalization and death after SARS-CoV-2 infection in the general population. The laboratory and hospital discharge databases of the Azienda Sanitaria Universitaria Friuli Centrale were used, and 5415 subjects infected with SARS-CoV-2 and with at least one recent absolute lymphocyte count determination before SARS-CoV-2 positivity were included. In total, 817 (15.1%) patients had severe COVID-19. Patients developing severe COVID-19 were more frequently males (44.9% of the severe COVID-19 group vs. 41.5% in the non-severe COVID-19 group; *p* < 0.0001) and were older (73.2 ± 13.8 vs. 58.4 ± 20.3 years; *p* < 0.0001). Furthermore, 29.9% of the lymphopenic patients developed severe COVID-19 vs. 14.5% of the non-lymphopenic patients (*p* < 0.0001). In a logistic regression model, female sex remained a protective factor (OR = 0.514, 95%CI 0.438–0.602, *p* < 0.0001), while age and lymphopenia remained risk factors for severe COVID-19 (OR = 1.047, 95%CI 1.042–1.053, *p* < 0.0001 for each additional year of age; OR = 1.715, 95%CI 1.239–2.347, *p* = 0.0011 for lymphopenia). This provides further information to stratify the risk of COVID-19 severity, which may be an important element in the management of immunosuppressive therapies.

## 1. Introduction

Since the SARS-CoV-2 outbreak began, there has been a great deal of interest in finding the risk factors for severe COVID-19. This knowledge, in fact, may guide policymakers in making decisions about non-pharmacological interventions, such as mitigation strategies, may provide insight into COVID-19 pathophysiological mechanisms and help rule out potential confounders in clinical trials [1].

In this context, the definition of possible risks for immunosuppressed patients has been a major issue, mainly due to the clinical challenge of immunosuppressive therapy management, which has been addressed by several scientific societies and group studies [2,3,4]. Immunocompromized patients represent a population in which respiratory viruses are an important cause of morbidity and mortality [5], and some evidence points to a more severe course and a higher risk of superimposed bacterial infections after SARS-CoV-2 infection in these patients [6]. On the other hand, severe COVID-19 is characterized by systemic hyper-inflammation described as cytokine storm, and this observation led to the study and clinical utilization of several anti-inflammatory and immune-suppressant agents [7]. Dexamethasone was the first drug to show a mortality benefit in clinical trials [8], followed by tocilizumab [9], an IL-6 receptor blocker, and baracitinib [10], a Janus kinase inhibitor. These observations raise the question as to whether immunosuppressed patients have more severe COVID-19 or, on the contrary, they are protected from cytokine storm and, therefore, severe disease [11]. Considering specifically lymphopenic patients, they present an increased risk of hospitalization with infection, pneumonia, and infection-related death with respect to the general population [12]. Lymphopenia is also a feature of COVID-19, being present in 35–75% of cases [13], and patients who become lymphopenic due to COVID-19 present worse outcomes [13,14,15]. However, little is known about the impact of previously existing lymphopenia on disease severity. The aim of this study was to determine, in the general population, if patients who were lymphopenic prior to SARS-CoV-2 infection presented a different risk of severe COVID-19.

## 2. Materials and Methods

We conducted a retrospective cohort study using the health administrative databases of the Azienda Sanitaria Universitaria Friuli Centrale in the 530,000-inhabitant province of Udine in north-eastern Italy. The laboratory and the hospital discharge databases were used. They are anonymous but can be linked to each other at the individual patient level through a stochastic key, which is univocal for each patient in both databases.

We included subjects with a positive nasopharyngeal swab real-time reverse-transcriptase assay for SARS-CoV-2 from 1 March 2020 to 31 December 2020 and at least one absolute lymphocyte count (ALC) determination between 180 and 14 days before SARS-CoV-2 positivity. In cases of more than one determination, the most recent one was considered in the analysis. We did not consider ALC measured in the 14 days before SARS-CoV-2 positivity due to the already considered ALC effect on COVID-19 severity [13,14,15]. Patients who required hospitalization or died were considered as having severe COVID-19, while patients who survived and did not require hospitalization were considered as having non-severe COVID-19.

We defined subjects as lymphopenic if they presented ALC < 800/μL, following Common Terminology Criteria for Adverse Events (CTCAE v5.0). Statistical analysis was performed using SAS v9.4 (SAS Institute Inc., Cary, NC, USA). Characteristics were compared between different outcome groups using the Wilcoxon two-sample Rank Sums test for continuous variables (age and lymphocyte count) and a chi-squared test for categorical ones (sex and lymphopenia). The Kolmogorov–Smirnov test with Lilliefors significance correction was used to assess the normal distribution of data. We used a logistic regression model to adjust for the potentially confounding effect of age and sex on the association between disease severity and lymphocyte count. Odds ratios (ORs) and 95% confidence intervals (95% CI) were calculated. We considered two-tailed *p*-values of <0.05 statistically significant.

## 3. Results

**Figure 1:**
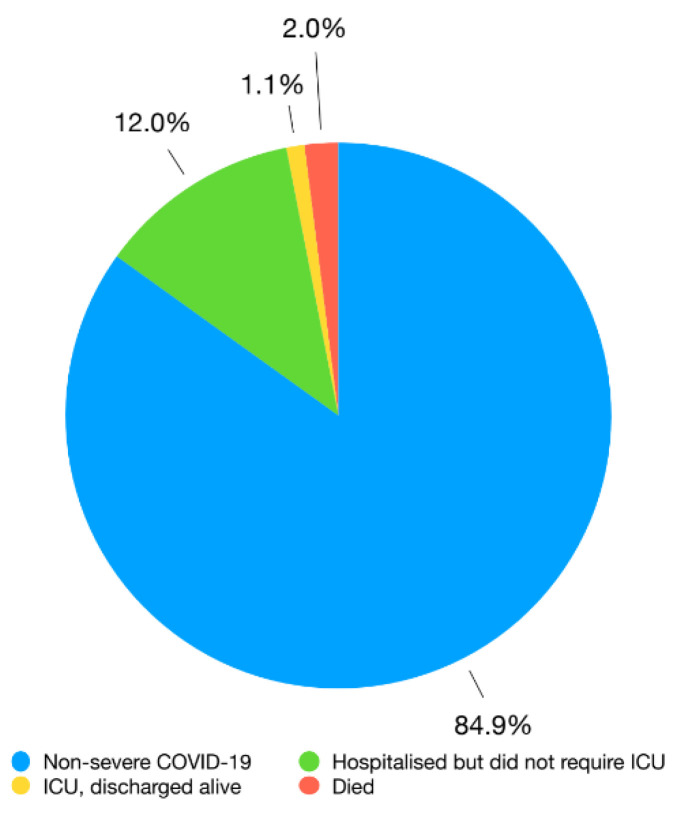
COVID-19 patients’ outcomes. ICU = intensive care unit.

We identified 5415 subjects, 2357 males (43.5%) and 3058 females (56.5%), with a mean age of 60.6 ± 20.1 years (median 62). The majority of the patients (4598; 84.9%) survived the infection and did not require hospitalization, while 817 (15.1%) patients had severe COVID-19. Furthermore, 108 patients died (2.0%), 58 had an intensive care unit (ICU) stay but were discharged alive (1.1%), and 651 were hospitalized in other wards and discharged alive (12.0%), as shown in Figure 1.

Patients developing severe COVID-19 were more frequently males (44.9% of the severe COVID-19 group vs. 41.5% in the non-severe group; *p* < 0.0001) and were significantly older (73.2 ± 13.8 (median 76) vs. 58.4 ± 20.3 (median 58) years; *p* < 0.0001).

In the overall population, 204 patients were lymphopenic (3.8%). In total, 61 lymphopenic patients developed severe COVID-19 (29.9% vs. 14.5% in non-lymphopenic patients, *p* < 0.0001). In particular, the ALC was lower in patients with severe disease (1907/μL ± 2727, median 1620 vs. 1949/μL ± 3001, median 1790; *p* < 0.0001). Table 1 summarizes the main differences between the severe and non-severe COVID-19 groups. In a logistic regression model, female sex remained a protective factor (OR = 0.514, 95%CI 0.438–0.602, *p* < 0.0001), while age and lymphopenia remained risk factors for severe COVID-19 (OR = 1.047, 95%CI 1.042–1.053, *p* < 0.0001 for each additional year of age; OR = 1.715, 95%CI 1.239–2.347, *p* = 0.0011 for lymphopenia).

## 4. Discussion

Since the beginning of the SARS-CoV-2 pandemic, different risk factors for severe COVID-19 have been described, including older age, male sex, and comorbidities, such as hypertension, diabetes mellitus [15] and obesity [16]. Lymphopenia is associated with the risk of infections [12], cardiovascular disease, cancer, liver disease, and systemic autoimmune disease [17]. Recently, lymphopenia has been described to be associated with reduced longevity independently of age, clinical risk factors, and other immunohematologic parameters [18], and in the general population, it is associated with a 1.6-fold increase in all-cause mortality [17].

Data regarding the effect of previously existing lymphopenia on COVID-19 are scarce and come mainly from selected populations of immunosuppressed patients. Chronic lymphopenia or corticosteroid utilization have been proven to be associated with hospitalization but not with severe respiratory illness in patients with cancer [19]. In a study on multiple sclerosis patients, lymphopenia not due to fingolimod treatment before SARS-CoV-2 infection was more frequent in more severe infections [20]. In a study on 51 HIV-infected individuals, CD4 cell counts of less than 200 cells/μL prior to SARS-CoV-2 infection were not significantly related with the clinical characteristics, treatments, or outcomes [21]. Our research suggests that, as for other infectious diseases, lymphopenic patients are at a higher risk of hospitalization and death secondary to SARS-CoV-2 infection.

Different mechanisms may explain our findings. Lymphopenia may affect T cell-mediated immune responses, with consequent delayed viral elimination [22]. The presence of viral load assessed with quantitative polymerase chain reaction in the lungs of patients at autopsy supports the inability to eliminate the pathogen as a possible mechanism [23]. A reduction in Treg cells may also cause a diversion of the adaptive immune responses towards innate-mediated inflammatory responses, with the hyperactivation of macrophages and neutrophils and cytokine storm, which ultimately leads to multi-organ failure and death [22]. Lymphocyte depletion may also cause general immunosuppression in COVID-19 patients [22], exposing them to superimposed infection. However, the relative importance of the proposed mechanisms, or the presence of alternative ones, has yet to be clarified.

Since these considerations have been made for lymphopenia caused by COVID-19, an additional effect determined by previously existing lymphopenia may be advocated. Interestingly, an open-label, multicentre, randomized clinical trial evaluating recombinant human granulocyte colony-stimulating factor (rhG-CSF) was conducted on lymphopenic COVID-19 patients. In this study, the rhG-CSF treatment led to the rapid restoration of lymphocyte cell counts and appeared to decrease the frequency of patients progressing to critical illness or death [24], thus supporting the important role of lymphopenia in determining COVID-19 severity.

The present study is clearly limited by its observational and retrospective nature, and confounding effects from unobserved factors may have affected our results. In particular, lymphopenia causes and durations were not taken into account, and it is known that lymphopenia may reflect adverse inflammatory, metabolic, or neuroendocrine stressors and thus may be associated with reduced survival as an epiphenomenon [18]. Older age is known to be associated with lower ALC [17] and COVID-19 severity [15], but we controlled for this confounding factor in the logistic regression. However, we did not consider other comorbidities that may have an effect on COVID-19 severity, and we also did not examine eventual differences in the treatment between the two groups.

## 5. Conclusions

The present study suggests that pre-existing lymphopenia may lead to more severe COVID-19. This provides further information to stratify the risk of COVID-19 severity in programming interventions of public health and may be an important factor in the management of immunosuppressive therapies.

## Figures and Tables

**Table 1:** Characteristics of non-severe and severe COVID-19 patients. N = number; SD = standard deviation; ALC = absolute lymphocyte count.

	Non Severe COVID-19	Severe COVID-19	*p*-Value
Male sex (N; %)	1907; 41.5%	450; 44.9%	<0.0001
Age (mean ± SD)	58.4 ± 20.3	73.2 ± 13.8	<0.0001
Lymphopenia (N; %)	143; 3.1%	61; 7.5%	<0.0001
ALC (mean ± SD)	1949/μL ± 3001	1907/μL ± 2727	<0.0001
Total (N; %)	4598; (84.9%)	817; (15.1%)	

## Data Availability

All data generated or analysed during this study are included in this published article.
